# Dexamethasone restores TNFα-induced epithelial barrier dysfunction in primary rat alveolar epithelial cells

**DOI:** 10.1371/journal.pone.0295684

**Published:** 2023-12-27

**Authors:** Naokata Kutsuzawa, Yoko Ito, Shizuko Kagawa, Chinatsu Kohno, Hiroto Takiguchi, Koichiro Asano

**Affiliations:** Division of Pulmonary Medicine, Department of Medicine, Tokai University School of Medicine, Isehara, Kanagawa, Japan; Eötvös Loránd Research Network Biological Research Centre, HUNGARY

## Abstract

Alveolar barrier dysfunction is one of the major pathophysiological changes in acute lung injury (ALI)/acute respiratory distress syndrome (ARDS). In ALI/ARDS, tumor necrosis factor-alpha (TNFα) disrupts the barriers of alveolar epithelium and endothelium. Glucocorticoids (GCs) exert anti-inflammatory effects and ameliorate pulmonary edema in ALI/ARDS. However, the involvement of GCs in the restoration of alveolar epithelial barrier dysfunction has not been extensively studied. Here, we elucidated that dexamethasone (Dex) restored TNFα-induced alveolar epithelial barrier dysfunction *in vitro* using primary rat alveolar epithelial cells isolated from Sprague–Dawley rats. Moreover, Dex promoted the alveolar epithelial cell barrier integrity by initiating GC receptor-mediated signaling *via* the downregulation of myosin light chain kinase (MLCK) expression and the dephosphorylation of myosin light chain (MLC) 2. Further investigation revealed that Dex enhanced the expression of zonula occludens-1 (ZO-1), a tight junction-related protein, at intercellular junction sites. These findings suggest that GCs strengthen the integrity of the alveolar epithelial barrier in ALI/ARDS *via* the GR-MLCK-pMLC2 axis.

## Introduction

Acute lung injury (ALI)/acute respiratory distress syndrome (ARDS) is caused by various diseases, including viral infections such as COVID-19, and exacerbation of interstitial pneumonia (IP) [1, 2]. In ALI/ARDS, various inflammatory cytokines are released, leading to barrier dysfunction in the alveolar epithelium and vascular endothelium. Subsequently, protein-rich exudates leak into the alveolar space, impairing gas exchange and resulting in hypoxemia and death [3].

The alveolar epithelium comprises type I and II alveolar epithelial cells. Type II alveolar epithelial (ATII) cells play crucial roles in innate immunity and the maintenance of the alveolar environment by producing pulmonary surfactants and replicating and differentiating into type I alveolar epithelial (ATI) cells. ATI cells are flat, elongated in shape, and cover 95–98% of the alveolar surface area, which enables gas exchange and maintains the alveolar barrier integrity [4, 5]. The barrier function of alveolar epithelial cells is regulated by tight junction (TJ)-related proteins, including the transmembrane proteins like claudins and occludin and the scaffolding protein zonula occludens (ZO) [6, 7]. Among various inflammatory cytokines produced in ALI/ARDS, tumor necrosis factor-alpha (TNFα) and interleukin (IL)-1β [3] alter TJ-related protein expression and impair the barrier function of the alveolar epithelium and vascular endothelium [8, 9].

Glucocorticoids (GCs) are widely used for the treatment of ALI/ARDS and related conditions such as COVID-19 pneumonia and acute exacerbation of IP. Although their use in clinical settings remains controversial [3], GCs exert therapeutic effects through their anti-inflammatory activities [10], and they ameliorate pulmonary edema *via* ion transport by the epithelial sodium channel (ENaC)/Na^+^-K^+^ ATPase/Na ^+^/H^+^ exchanger axis in ALI/ARDS [11]. GCs are also used as therapeutic agents for inflammatory bowel disease (IBD) [12]; the pathogenesis of IBD involves intestinal epithelial barrier dysfunction [13]. Previous studies have revealed that GCs improved intestinal barrier dysfunction by altering TJ-related protein expression in TNFα-induced models mimicking IBD [14–16]. However, little is known whether GCs ameliorate alveolar epithelial barrier dysfunction, and the underlying mechanisms are unclear.

The perijunctional rings of actin and myosin II support the TJs. Moreover, myosin light chain kinase (MLCK) plays an important role in the regulation of the perijunctional actomyosin ring via phosphorylation of myosin light chain (pMLC) and modifies physiological and pathophysiological barrier regulation [17]. ZO-1 binds to actin and is stabilized at the TJs following MLCK inhibition [18]. Therefore, the interaction between ZO-1 and actin, which is regulated by the MLCK-pMLC axis, is crucial for TJ permeability.

Therefore, we hypothesized that GCs could restore alveolar epithelial barrier dysfunction during the acute phase of ALI/ARDS by altering the expression of TJ-related proteins. In this study, we examined the effect of dexamethasone (Dex) on TNFα-induced barrier dysfunction model of rat primary alveolar epithelial cells *in vitro*.

## Materials and methods

### Reagents

Recombinant rat TNFα (rTNFα) was purchased from R&D System Inc. (Minneapolis, MN, USA). Dex was purchased from Sigma-Aldrich (St. Louis, MO, USA). RU-486 (mifepristone), a GC receptor blocker, was supplied by Cayman Chemical Company (Ann Arbor, MI, USA). The ENaC blocker amiloride was purchased from Abcam (Cambridge, UK). All the chemicals used were analytical grade.

### Ethics committee approval

All experimental procedures and protocols involving animals were reviewed and approved by the Institutional Animal Care and Use Committee of the Tokai University (Protocol Number which were reviewed and renewed annually: 203006, 214003, 225005, 231002), and were performed in compliance with the relevant guidelines and regulations. The method of euthanasia for animals is not inconsistent with American Veterinary Medical Association (AVMA) Guidelines for the Euthanasia of Animals 2020.

### Rat ATII cell isolation

ATII cells were isolated from pathogen-free, adult (6-week-old), male Sprague–Dawley rats (weight: 240–350 g; Charles River Laboratories Japan, Inc., Yokohama, Kanagawa, Japan). Briefly, the rats were euthanized using overdosages of pentobarbital (100 mg/body) by intraperitoneal injection. After euthanasia, the chest was opened, and lungs were perfused and removed. Then, lungs were incubated with porcine pancreatic elastase (Worthington, Lakewood, NJ, USA) for dissociation, and cells were purified on discontinuous density gradient according to a previously described method [19]. Finally, the freshly isolated cells were kept in 10% dimethyl sulfoxide (DMSO) and 90% fetal bovine serum (FBS) and frozen in liquid nitrogen until further use.

### Primary rat ATII cell culture and development of ATI-like cell monolayer

**Fig 1:**
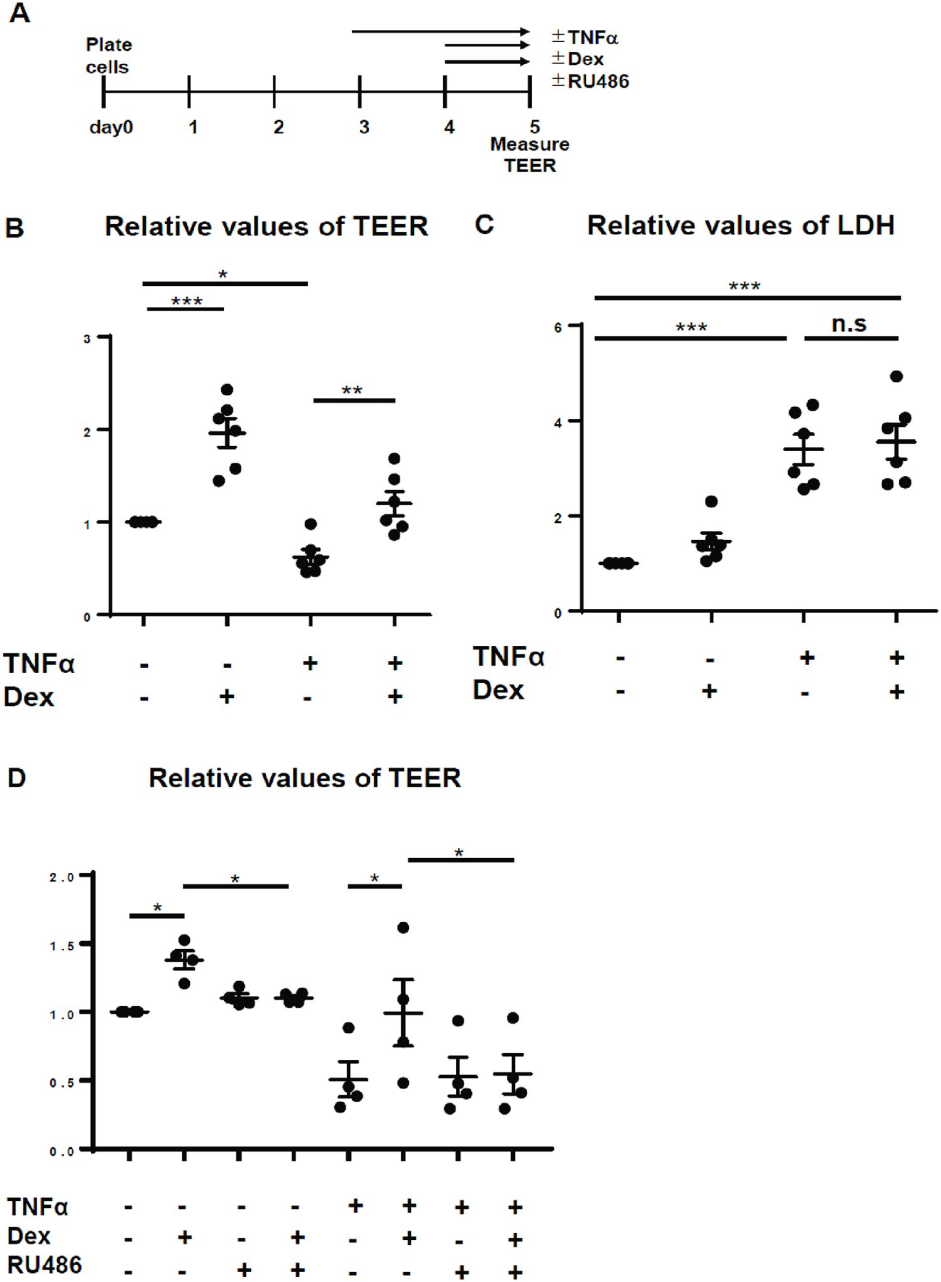
Effect of TNFα, Dex, and RU486, a glucocorticoid receptor antagonist, on TEER and cytotoxicity in alveolar epithelial cells (AECs). (A) Experimental protocol (B) Relative values of TEER in AECs stimulated with ± 10 ng/mL recombinant rat TNFα for 48 h and ± 1,000 nM Dex for 24 h. (C) Cytotoxicity was measured using the LDH assay at the same time point as measurement of TEER, n = 6 (six independent experiments, triplicate wells/experiment, Each dot means the average of triplicate wells.); (D) Relative values of TEER in AECs under treatment of ± 10 ng/mL recombinant rat TNFα for 48h, ± 1000 nM Dex and ± 10 μM RU486 for 24 h, n = 4 (four independent experiments, triplicate wells/experiment, Each dot means the average of triplicate wells.); values are expressed as mean ± standard error of the mean; * p<0.05, ** p<0.01, *** p<0.001; TEER: transepithelial electrical resistance, TNFα: tumor necrosis factor-alpha, Dex: dexamethasone, n.s.: not significant.

We resuspended frozen rat ATII cells (1 × 10^6^ cells/insert) in Dulbecco’s minimal essential medium (DMEM: Gibco Thermo Fischer Scientific, Waltham, MA, USA). Next, for differentiation into ATI-like cells, the ATII cells were cultured in rat tail collagen I (Gibco Thermo Fischer Scientific)-coated 12-well transwell inserts (MCRP12H48: area 1.1 cm^2^, pore size 1.0 μm, 0.91×10^6^ cells/cm^2^; Millicell Hanging Cell Culture Inserts, EMD Millipore Corporation. Billerica, MA, USA) in 0.5 mL of DMEM supplemented with 10% FBS. The basal side of the tissue culture plates was filled with 1.5 mL of the same medium as that on the apical side. The cells were cultured in DMEM supplemented with 10% FBS for 1 d, then cultured in DMEM supplemented with 5% FBS for 2 d to develop an ATI-like cell monolayer [20]. On day 3, the apical and basal sides of the medium were changed to FBS-free DMEM ± 10 ng/mL rTNFα (first stimulation). Twenty-four hours after the first stimulation using rTNFα, the culture medium was changed to FBS-free DMEM ± 1,000 ng/mL Dex, ±10 ng/mL rTNFα, or ± 1 μM RU486 (Fig 1A). All experiments were repeated three to five times independently, and each independent experiment was done by triplicate wells/condition.

### Transepithelial electrical resistance (TEER)

The barrier integrity of the alveolar epithelial cell monolayer was assessed by measuring TEER using a Millicell ERS-2 voltmeter (EMD Millipore Corporation). TEER was measured twice for each Transwell, and the average values were calculated for analysis.

### Real-time RT-PCR

Cells were lysed in Buffer RLT (Qiagen, Hilden, Germany), and RNA was purified using the RNeasy Mini QIA Kit (Qiagen) according to the manufacturer’s instructions. For real-time RT-PCR, the gene expression levels were normalized to that of the constitutive probe peptidylprolyl isomerase A (*Ppia*). Specific primers and probes for *ZO-1*, *MLCK*, and *ENaCα*, *β*, and *γ* subunits, claudin18 (*CLDN18*) and occludin (*OCLN*) were purchased from Applied Biosystems (Waltham, MA, USA). qPCR was performed for 40 cycles on the Cobas Z (Roche Diagnostics, Basel, Switzerland). Relative mRNA expression levels were calculated using the 2^−ΔΔCt^ method.

### Immunoblotting

Protein expression in ATI-like cells was measured using the Simple Western System Wes (ProteinSimple, Inc., San Jose, CA, USA) according to the manufacturer’s instructions. Rabbit anti-ZO-1 and anti- phospho-myosin light chain 2 (pMLC2) antibodies were purchased from Thermo Fisher Scientific and Cell Signaling Technology (Danvers, MA, USA), respectively. Briefly, the proteins were electrophoretically separated according to their molecular weight to create a ladder for capture of reactive antibodies. For the secondary antibody, ready to use horseradish peroxidase (HRP)‐conjugated goat anti‐rabbit antibody (ProteinSimple, Inc.) was used. Digital image of chemiluminescence of the capillary was captured with the Compass Simple Western software (ProteinSimple, Inc.), that automatically calculated chemiluminescence intensity of each single antigen binding signal. Results could be visualized as electropherograms representing peak of chemiluminescence intensity and as lane view from signal of chemiluminescence detected in the capillary. Protein loading was normalized to that of rabbit anti-glyceraldehyde-3-phosphate dehydrogenase (GAPDH) (St. Louis, MO, USA).

### Lactate dehydrogenase (LDH) assay

To assess toxicity in ATI-like cells, the LDH levels in the culture medium were measured using the cytotoxicity LDH assay kit (Dojindo Molecular Technology, Inc., Kumamoto, Japan) according to the manufacturer’s instructions.

### Immunocytochemistry

To detect ZO-1 and phalloidin protein expression in ATI-like cells, they were cultured in a 12-well hanging insert and fixed using 4% paraformaldehyde. After blocking with 3% normal goat serum (Rockland Immunochemicals Inc., Limerick, PA, USA) in PBS, the cells were incubated overnight with rabbit anti-ZO-1 antibody. Next, the cells were incubated with the secondary antibody Alexa Fluor 488 goat anti-rabbit IgG (Invitrogen, Carlsbad, CA, USA) for 1 h. Subsequently, the cells were mounted in Vectashield medium containing 4’,6-diamidino-2-phenylindole (DAPI) (Vector Laboratories, Burlingame, CA, USA).

The images of ZO-1 immunostaining were taken from two areas randomly in each condition of each experiment (n = 6). Tight junctions show normally linear structures when observed with immunofluorescence microscopy. However, tight junctions are also adapted to non-linear architectures such as either a ruffled or spiked morphology, with responding to changes in claudin engagement of actin filament [21]. Therefore, to evaluate the link between ZO-1 localization and actin organization, the number of spikes were counted from a central cell of each image and cells bordering it.

### Statistical analysis

Student’s *t*-test was performed using Graphpad Prism 8.0 to evaluate statistical differences between two experimental groups. One-way ANOVA was performed using GraphPad Prism 8.0 to evaluate statistical differences between more than three experimental groups. Fisher’s least significant difference test and Tukey’s multiple comparison test were used for multiple comparisons, and the results were considered statistically significant at p <0.05. All data are shown as mean±standard error of the mean.

## Results

### Effect of Dex on TEER

We stimulated the rat AT1-like epithelial cell monolayer using 10, 100, and 1,000 nM dexamethasone (Dex) and observed a dose-dependent increase in TEER, which plateaued at 100 nM Dex treatment (S1A Fig). Moreover, a time-dependent increase in TEER was observed after 6 h of Dex administration, which persisted for 24 h. However, the Dex-induced increase in TEER disappeared by 48 h (S1B Fig).

### Effect of TNFα and Dex on TEER and cytotoxicity

We stimulated the rat ATI-like epithelial cell monolayer using 10 ng/mL rTNFα, which induced a decrease in TEER after 24 (S2A Fig) and 48 h (Fig 1B and S2B Fig). Subsequently, 1,000 nM Dex was added to the epithelial cell monolayer after 24 h of TNFα exposure. The TNFα-induced decline in TEER was restored by Dex after 24 h of its administration (Fig 1B). The LDH assay revealed that TNFα-induced cytotoxicity was not alleviated by Dex treatment (Fig 1C), suggesting that the Dex-induced increase in TEER was not due to the inhibition of cytotoxicity.

### Effect of GC receptor inhibition on TEER

To examine the role of GC receptor (GR) on the Dex-induced increase in TEER, the epithelial cell monolayer was treated using 10 μM RU486, a GR inhibitor, along with Dex after 24 h of TNFα treatment. The schematic experimental protocol was shown in Fig 1A. RU486 abrogated the Dex-induced increase in TEER regardless of the presence of rTNFα (Fig 1D and S2C Fig). These results suggested that the Dex-induced increase in TEER occurred via the GR signaling pathway.

### Effect of Dex on ENaC mRNA expression and the effect of Dex-induced ENaC expression on TEER

**Fig 2:**
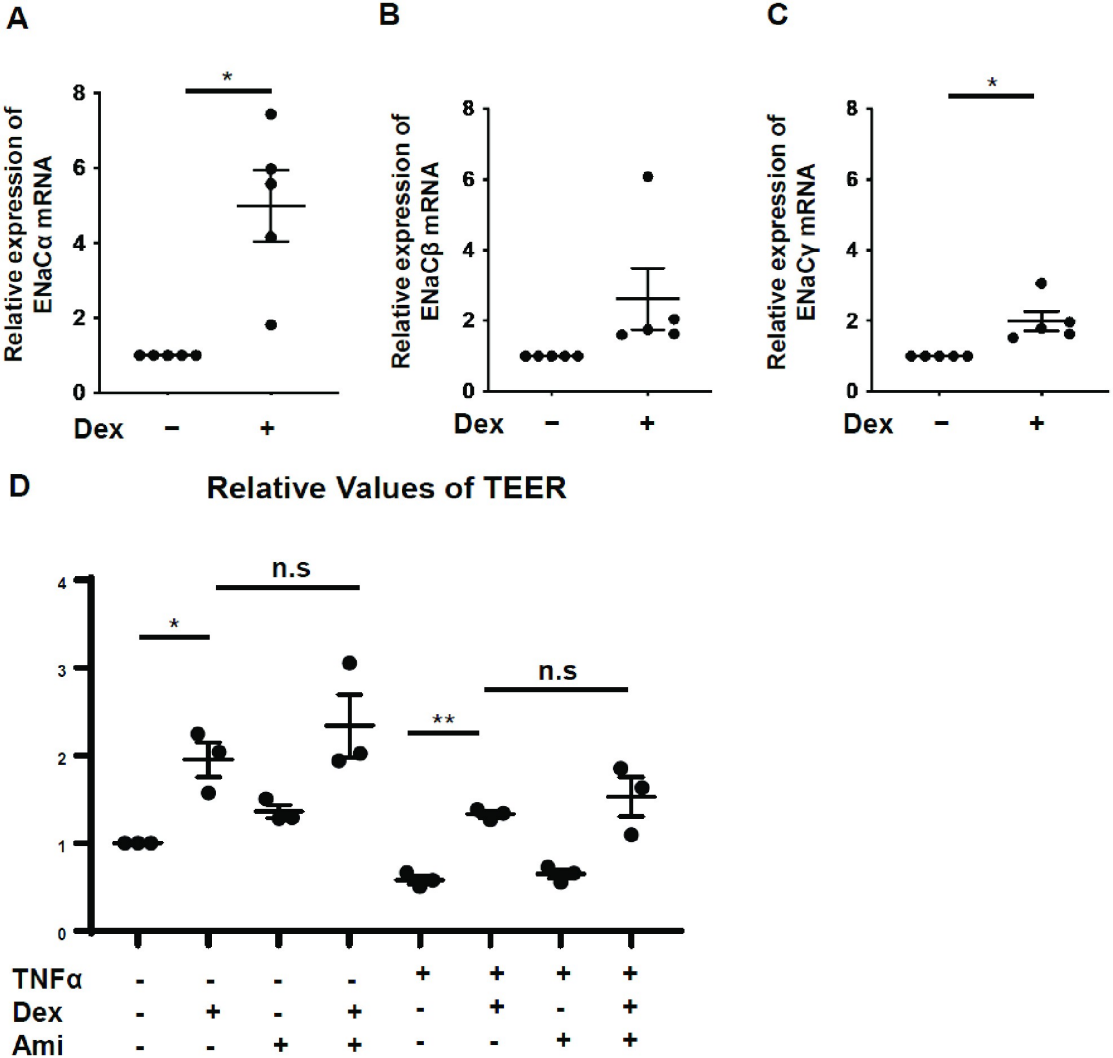
Effect of Dex on ENaC mRNA expression and the effect of Dex-induced ENaC expression on TEER. (A, B, C) Relative expression of ENaCα, ENaCβ, and ENaCγ mRNA in AECs after 6 h of Dex stimulation, n = 5 (five independent experiments); (D) Relative values of TEER in AECs stimulated using ± 10 ng/mL recombinant rat TNFα ± 1,000 nM Dex ± 10 ng/mL Amiloride after 24 h of Dex exposure, n = 3 (three independent experiments, triplicate wells/experiment, Each dot means the average of triplicate wells.); values are expressed as mean ± standard error of the mean; * p<0.05, ** p<0.01; ENaC: epithelial sodium channel, TEER: transepithelial electrical resistance, TNFα: tumor necrosis factor-alpha, Dex: dexamethasone, Ami: amiloride, n.s.: not significant.

We confirmed that the Dex-induced ENaC expression in alveolar epithelial cells was similar to that reported in previous studies [22, 23]. The upregulation of ENaCα and γ subunit mRNA levels, except for that of β the subunit, was statistically significant (Fig 2A–2C). Sodium ion absorption by epithelial cells increases due to upregulated ENaC on the apical cell membrane, which is actively transported to the interstitium by basolateral Na^+^-K^+^ ATPase [24, 25], would increase TEER. To explore the effect of ENaC on the Dex-induced increase in TEER, the epithelial cell monolayer was stimulated using 10 ng/mL amiloride, an ENaC inhibitor, along with Dex for 24 h in the presence or absence of TNFα treatment. Amiloride did not alter the Dex-induced increase in TEER (Fig 2D and S3 Fig), suggesting that TEER was not affected by ENaC activity.

### Effect of Dex on ZO-1 expression

**Fig 3:**
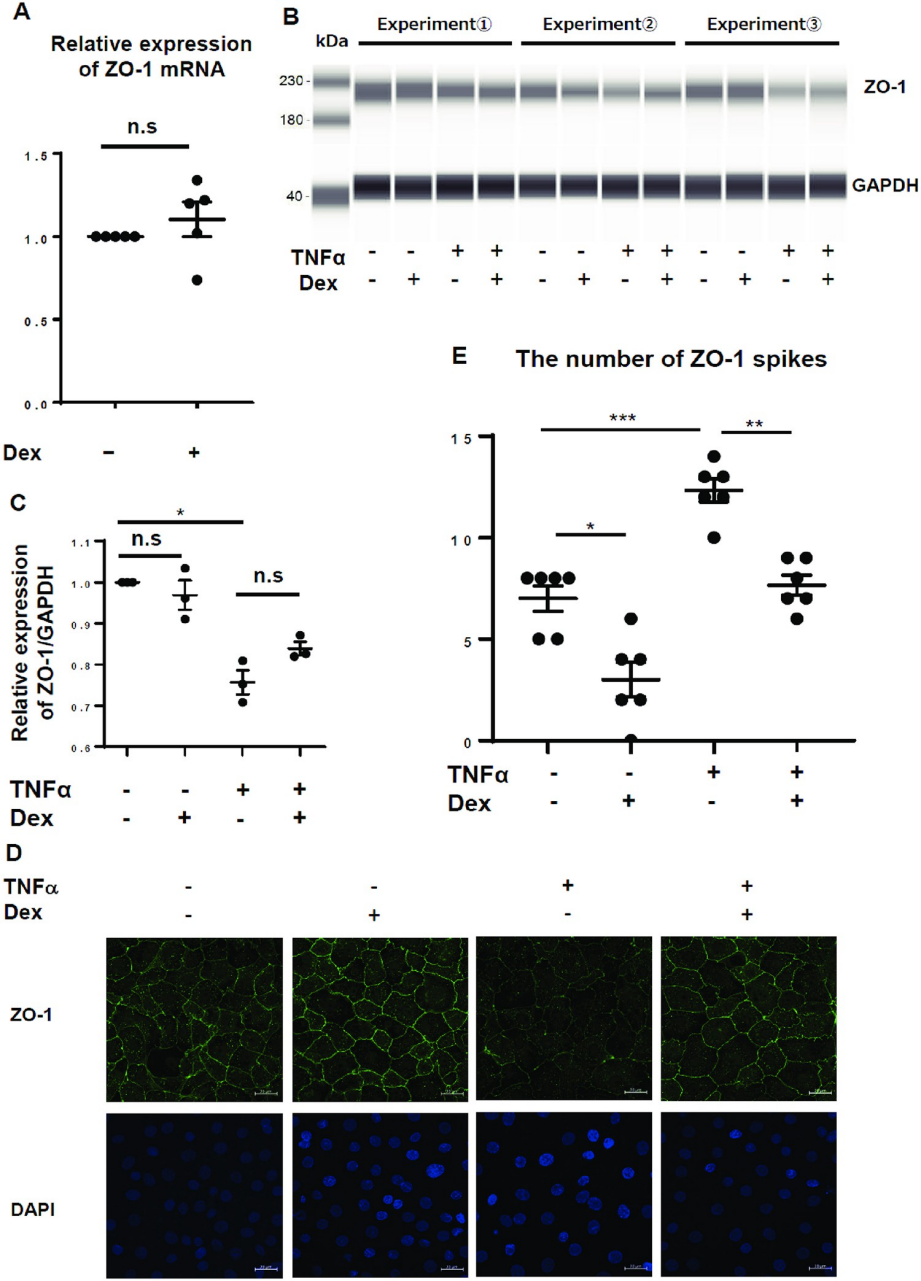
Effect of TNFα and Dex on ZO-1 expression. (A) Relative expression of ZO-1 mRNA in AECs after 6 h of Dex stimulation, n = 5 (five independent experiments); (B) Protein levels of ZO-1 (normalized to those of GAPDH) were measured using immunoblotting, n = 3 (three independent experiments); (C) Relative intensity of ZO-1 after 24 h of Dex stimulation assessed using immunoblotting analysis of three independent experiments; (D) Representative immunostaining of AEC monolayer after 24 h of Dex stimulation; green: ZO-1, blue: DAPI; magnification: ×400; (E) The number of ZO-1 spikes in immunostaining of AEC monolayer, n = 6 (six independent experiments); values are expressed as mean ± standard error of the mean; * p<0.05, ** p<0.01, *** p<0.001; ZO-1: zonula occludens-1, GAPDH: Glyceraldehyde-3-phosphate dehydrogenase, TNFα: tumor necrosis factor-alpha, Dex: dexamethasone, n.s.: not significant.

To determine the effect of Dex on TJ-related proteins, the expression of ZO-1, a TJ-related protein connecting the transmembrane proteins (e.g., claudins and occludin) and the actin cytoskeleton [26] was investigated. However, Dex treatment did not affect ZO-1 mRNA expression (Fig 3A). Moreover, immunoblotting analysis elucidated that ZO-1 protein levels were not affected by Dex treatment, although they were significantly reduced by TNFα treatment (Fig 3B and 3C and S4A and S4B Fig). Next, we examined the effect of Dex treatment on the intensity and distribution of ZO-1 in cells using immunofluorescence staining. TNFα treatment attenuated the expression of ZO-1 protein uniformly in the cells, whereas Dex treatment increased ZO-1 protein significantly at the intercellular junction sites (Fig 3D and S5 Fig). To evaluate the link between ZO-1 localization and actin organization, the number of spikes in images of ZO-1 immunostaining were compared (Fig 3E). TNFα treatment significantly increased the number of spikes, while Dex treatment reduced it.

### Effect of Dex on MLCK and pMLC2 levels

**Fig 4:**
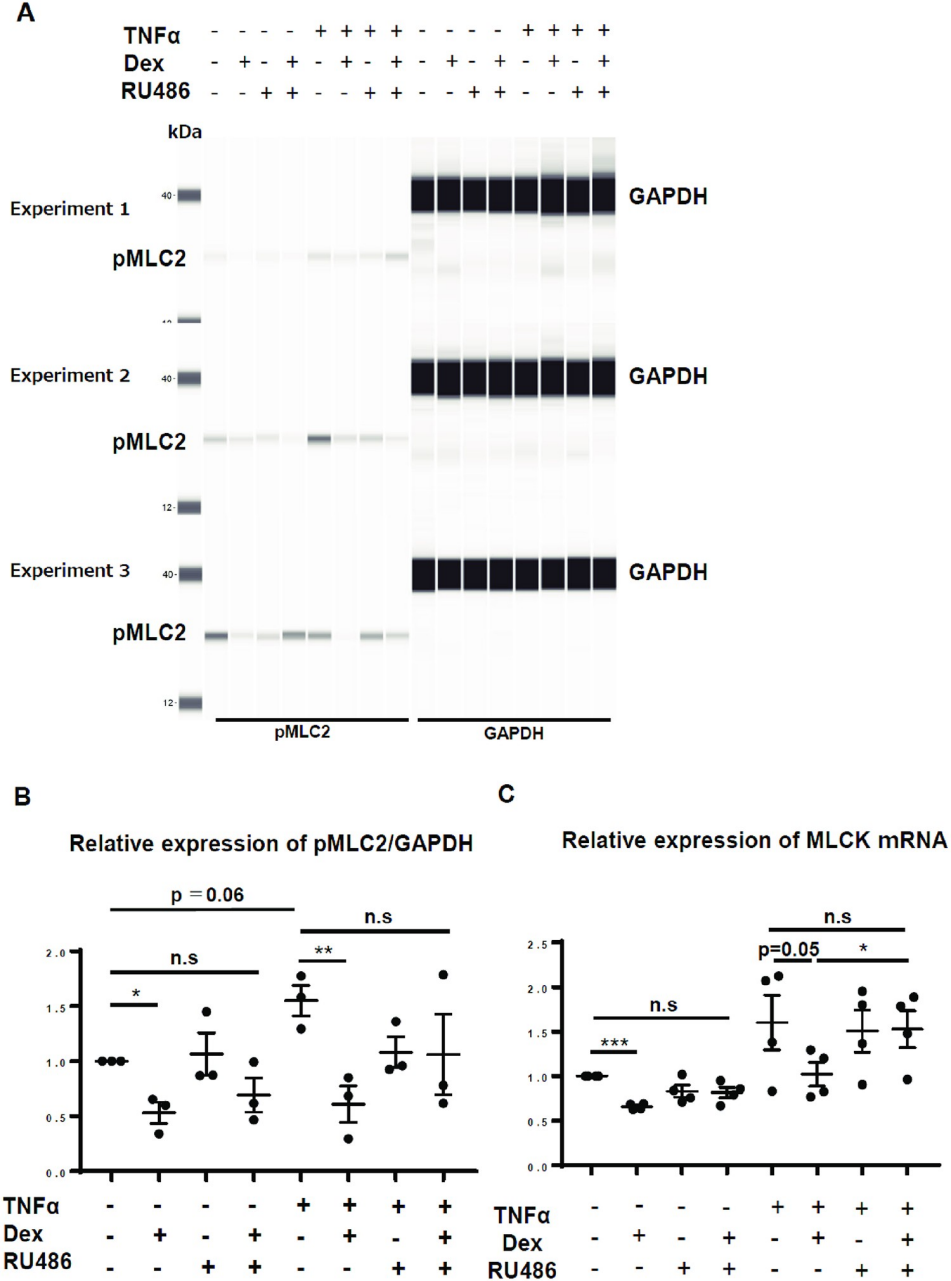
Effect of Dex on the expression of pMLC2 and MLCK. (A) Protein levels of pMLC2 (normalized to those of GAPDH) were measured using immunoblotting after 24 h of Dex exposure, n = 3 (three independent experiments); (B) Relative intensity of pMLC2 after 24 h of Dex stimulation, from immunoblotting analysis of three independent experiments; (C) Relative expression of MLCK mRNA in AECs after 6 h of Dex stimulation, n = 4 (four independent experiments); values are expressed as mean ± standard error of the mean; * p<0.05, *** p<0.001; pMLC2: phospho-myosin light chain 2, GAPDH: Glyceraldehyde-3-phosphate dehydrogenase, n.s.: not significant, MLCK: myosin light-chain kinase, TNFα: tumor necrosis factor-alpha, Dex: dexamethasone.

To further understand the effect of Dex on epithelial barrier function, we investigated the levels of pMLC2, which regulates actin contraction and relaxation, for the determination of the strength of epithelial barrier function [27, 28]. Dex treatment significantly suppressed pMLC2 levels (Fig 4A and 4B and S6A and S6B Fig). The mRNA levels of MLCK, a regulator of MLC2 phosphorylation, were also reduced following Dex treatment (Fig 4C). The suppression of pMLC2 and MLCK levels by Dex was abrogated by RU486 (Fig 4A–4C).

## Discussion

**Fig 5:**
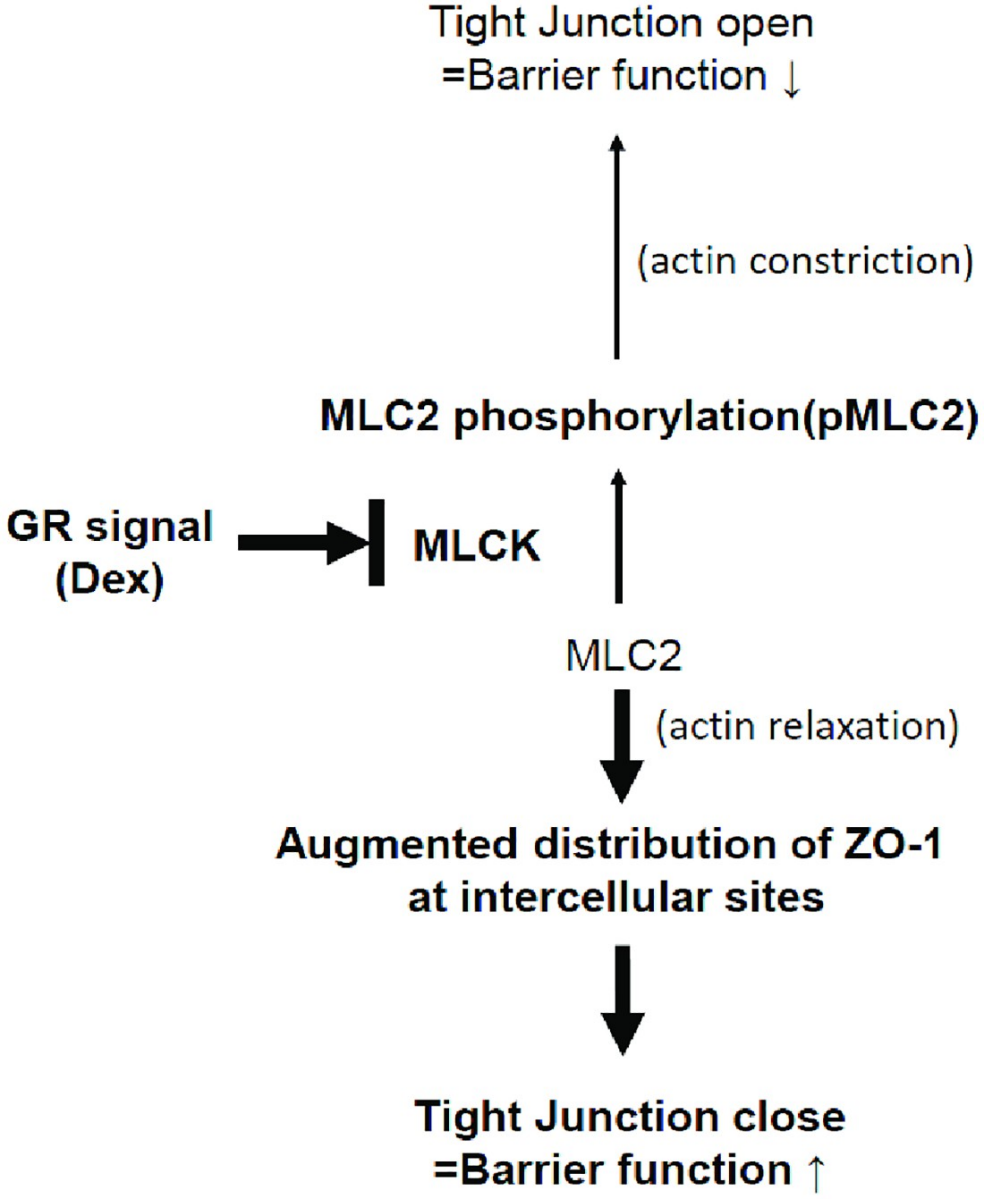
Schematic diagram representing glucocorticoid-induced protection of alveolar epithelial cell integrity via the regulation of the GR/MLCK/pMLC2 signaling pathway. GR, glucocorticoid receptor; Dex, dexamethasone; pMLC2, phospho-myosin light chain 2; MLCK, myosin light chain kinase, ZO-1: zonula occludens-1.

In this study, we elucidated that Dex treatment restored TNFα-induced alveolar epithelial barrier dysfunction by initiating GR-mediated signaling. Dex treatment also led to the downregulation of MLCK expression, dephosphorylation of MLC2, and increase in ZO-1 expression at the intercellular junction sites without significant changes in intracellular ZO-1 protein levels. Therefore, Dex treatment enhances the alveolar epithelial barrier function by altering the distribution of ZO-1, which could be via the MLCK/pMLC2 axis, leading to strengthen barrier function (Fig 5).

The ability of GCs to enhance the intestinal epithelial barrier function has been widely studied [14–16]. In Crohn’s disease, an IBD, TNFα disrupts the intestinal epithelial barrier function [13]. Boivin *et al*. reported that GCs restored the TNFα-induced intestinal epithelial barrier dysfunction by upregulating MLCK expression *via* a GR-mediated signaling pathway [15]. Moreover, Xu *et al*. demonstrated that TNFα impaired epithelial barrier function in the organoids generated using cells from patients with Crohn’s disease, which was rescued by prednisolone [16]. In their study, TNFα enhanced the levels of pMLC2, a downstream molecule of MLCK, followed by TJ impairment with elevated claudin-2 and decreased E-cadherin levels, whereas the GR signaling pathway induced MLC2 dephosphorylation leading to the restoration of TJ activity [16]. Therefore, the results of these studies on intestinal epithelial cells support our findings, suggesting that GCs regulate the alveolar epithelial barrier function *via* the GR-MLCK-pMLC2 axis.

However, in contrast to the aforementioned studies on intestinal epithelial cells, TNFα did not statistically induce MLCK expression and MLC2 phosphorylation in the present study. This difference may be due to the differences in cell types (cell line or primary, or species), doses of rTNFα, evaluated time-points, and culture methods. In the present study, we elucidated that TNFα induced cytotoxicity in alveolar epithelium and reduction in ZO-1 protein levels. Several studies have reported that TNFα reduces ZO-1 expression and inhibits ZO-1 recruitment to the intercellular spaces, thereby weakening TJ adhesion and impairing the barrier function of the airway, kidney, and intestinal epithelial cells [29–31]. These results support our findings and elucidate the underlying mechanisms of the TNFα-induced barrier dysfunction observed in our study.

ZO-1 directly binds to TJ proteins on the cell membrane and cross-links them to the actin cytoskeleton [26, 32]. A previous study demonstrated that ZO-1 was distributed and stabilized at the TJs following MLCK inhibition in Madin–Darby Canine Kidney (MDCK) cells [33]. Recently, a study reported that a 28-amino acid sequence in ZO-1 is responsible for actin binding and is essential for TJ permeability in MDCK cells [18]. Therefore, the accumulation of ZO-1 at the intercellular junction sites by Dex treatment in the current study suggests actin relaxation and concomitant alveolar TJ barrier closure. The speculation that Dex treatment enhances barrier function *via* actin-relaxation is supported by our findings that the alveolar barrier function was recovered by Dex treatment without the alleviation of TNFα-induced cytotoxicity or the enhancement of the decreased ZO-1 protein levels. Similar to previous studies, we attempted to directly quantify Dex-induced changes in the intracellular distribution of actin filament [34, 35] to verify our hypothesis. In our *in vitro* model, the direct quantification of actin relaxation and contraction with phalloidin immunostaining was difficult because of the lack of objectivity. However, as the molecular mechanisms that underlie tension generation by actin filaments and induce ruffles and spikes are suggested to be comparable (e.g. MLCK, Rho kinase activation) [21], we have quantified the number of spikes in ZO-1 to speculate the effect of TNFα and Dex treatment on actin tension. As shown in Fig 3E, the increased number of spikes by TNFα and decreased number by Dex indirectly suggest actin contraction and relaxation, respectively.

The phosphorylation of MLC2 leads to epithelial barrier dysfunction by inducing stress fiber formation, resulting in cell shrinkage [15, 17]. The RhoA/Rho-associated protein kinase (ROCK) signaling pathway is involved in the phosphorylation of MLC2 [36, 37]. Therefore, we investigated the effect of ROCK inhibitors on the alveolar epithelial barrier function; however, the effect on alveolar epithelial barrier function varied depending on the concentration of the ROCK inhibitor (Y27632: Cayman Chemical Co., Ann Arbor, MI., USA) (S7 Fig). Therefore, we did not investigate whether the RhoA/ROCK pathway is involved in the alveolar epithelial barrier function.

Some other possible mechanisms could also be involved in the recovery of the TNFα-induced alveolar epithelial barrier dysfunction by GCs. TEER has the potential to evaluate the physical barrier integrity between cell–cell junctions and to detect ion transfer through channels and transporters [25]. ENaCs are involved in the reabsorption and clearance of alveolar fluid when the alveolar barrier integrity, which prevents the leakage of fluid from the basal side into the alveolar space, is disrupted [22–25]. GCs enhance ENaC expression, which enhances the transfer of Na^+^ and other ions. Thus, to exclude the effect of ENaC on Dex-induced TEER, amiloride, an ENaC inhibitor, was administered along with Dex. However, amiloride did not affect TEER, suggesting that ENaC was not involved in the alteration of TEER by Dex.

Another possibility is the upregulated expression of TJ-related proteins, such as claudins and occludin. A previous study revealed that Dex promoted TNFα-induced alveolar epithelial barrier dysfunction by increasing TJ protein expression in A549 cells [38]. Another study reported that GCs upregulated the expression of the TJ protein claudin-8 in H441 cells, leading to an increase in TEER [39]. Although claudin-8 levels were not evaluated in our study, the mRNA and protein levels of occludin, a transmembrane protein of TJs, and claudin-18, a highly expressed in alveolar epithelial cells [7], were measured. However, these levels were not altered by Dex treatment in the present study (S8A and S8B Fig). This inconsistency could be because the previous studies used lung cancer cell-derived cell lines with different ultrastructure and phospholipid components and their suitability as an alveolar epithelial cell model is unclear [40].

This study has several limitations. First, the effect of GCs on the alveolar epithelial barrier function was studied only for a short duration because the TEER values in alveolar epithelial cells peaked at 4–5 d of culture, even under unstimulated conditions in our culture system (S1B Fig). This phenomenon made it difficult to understand the reason behind the enhancement of the barrier function by GCs for only 24 h. Therefore, it is unclear whether the short-term effect of the Dex-induced increase in TEER in this study is due to the longevity of the cultured cells or explaining the effect of GCs only observed in the acute phase of ALI/ARDS. Second, our study was performed using a monoculture of alveolar epithelial cells. In ALI/ARDS in a clinical setting, the disruption of both the epithelial and vascular endothelial barriers occurs, resulting in the leakage of inflammatory cells and protein-rich effluent into the alveolar space [3, 8, 9]. Recent evidence suggests that MLCK-mediated regulation of the barrier function in vascular endothelial cells is important as per an ARDS model in MLCK-knockout mice [41]. In contrast, in a model of lung injury caused by pneumoedematogenic gas inhalation, MLCK inhibitors improved lung injury by enhancing the alveolar epithelial cell barrier function [35]. Additionally, despite the use of TNFα in barrier dysfunction models in previous studies [8, 38], it is unlikely that TNFα alone contributes to the barrier dysfunction in clinical settings. Macrophages and other inflammatory cells in ALI/ARDS secrete various inflammatory mediators [3]. To validate the effect of GCs on the alveolar barrier function in ALI/ARDS, further investigation is required, using *in vivo* models or *in vitro* co-culture systems with macrophages, vascular endothelial, and alveolar epithelial cells. Finally, although our study revealed that Dex treatment downregulated MLCK expression and induced pMLC, these findings are descriptive because of lack of evidence for the determination of whether the effect of Dex on barrier function is abrogated by MLCK activation. Due to difficulties in genetically modifying primary cells, further studies using alveolar epithelial cells isolated from MLCK-overexpressing mice should be performed.

## Conclusions

We report, for the first time, that TNFα impaired the alveolar epithelial barrier function by inducing cytotoxicity and reducing ZO-1 protein expression in primary alveolar epithelial cells *in vitro*. We also suggest a novel molecular mechanism for the restoration of the barrier function by GCs, i.e., they act by downregulating MLCK expression and promoting the dephosphorylation of MLC2, which recruits the remaining ZO-1 proteins to the intercellular spaces. Therefore, these findings indicate that GCs can restore the alveolar epithelial barrier impairment in ALI/ARDS probably via the GR-MLCK-pMLC2 axis.

## Supporting information

S1 Fig Effect of Dex on barrier function in AECs. (A) TEER in AECs stimulated using different doses of Dex at 6 and 24 h; (B) Time course of TEER in AECs stimulated using 1,000 nM Dex; n = 1, triplicate wells/experiment, Each dot means TEER value from each well.; values are expressed as mean ± standard deviation; AECs: alveolar epithelial cells, TEER: transepithelial electrical resistance, Dex: dexamethasone.
(TIF)

S2 Fig Effect of TNFα on TEER at 24 h and effect of Dex and RU486, a glucocorticoid receptor antagonist, on TEER (not relative value) in alveolar epithelial cells (AECs). (A) TEER in AECs stimulated with ± 10 ng/mL recombinant rat TNFα for 24 h, n = 6 (six independent experiments). (B) TEER in AECs stimulated with ± 10 ng/mL recombinant rat TNFα for 48 h and ± 1,000 nM Dex for 24 h, n = 6 (six independent experiments). (C) TEER in AECs under treatment of ± 10 ng/mL recombinant rat TNFα for 48h, ± 1000 nM Dex and ± 10 μM RU486 for 24 h, n = 4 (four independent experiments); * p<0.05, ** p<0.01, *** p<0.001; TEER: transepithelial electrical resistance, TNFα: tumor necrosis factor-alpha, Dex: dexamethasone.
(TIF)

S3 Fig Effect of Dex-induced ENaC expression on TEER. TEER in AECs stimulated using ± 10 ng/mL recombinant rat TNFα ± 1,000 nM Dex ± 10 ng/mL Amiloride after 24 h of Dex exposure, n = 3 (three independent experiments, triplicate wells/experiment, Each dot means the average of triplicate wells.); values are expressed as mean ± standard error of the mean; ** p<0.01; TEER: transepithelial electrical resistance, TNFα: tumor necrosis factor-alpha, Dex: dexamethasone, Ami: amiloride, n.s.: not significant.
(TIF)

S4 Fig Digital images of chemiluminescence of the capillary captured with the Compass Simple Western software (ProteinSimple, Inc.), that automatically calculated chemiluminescence intensity of ZO-1 and GAPDH binding signal for Fig 3B and 3C. (A) Results visualized as electropherograms representing peak of chemiluminescence intensity and (B) as lane view from signal of chemiluminescence detected in the capillary.
(TIF)

S5 Fig Immunostaining of AEC monolayer after 24 h of Dex stimulation from six independent experiments (2 areas /experiment). green: ZO-1; magnification: ×400.
(TIF)

S6 Fig Digital images of chemiluminescence of the capillary captured with the Compass Simple Western software (ProteinSimple, Inc.), that automatically calculated chemiluminescence intensity of pMLC2 and GAPDH binding signal for Fig 4B and 4C. (A) Results visualized as electropherograms representing peak of chemiluminescence intensity and (B) as lane view from signal of chemiluminescence detected in the capillary.
(TIF)

S7 Fig Effect of ROCK inhibitor on barrier function in AECs. TEER in AECs stimulated using different doses of ROCK inhibitor (Y27632) at 24 h; n = 1, triplicate wells/experiment, Each dot means TEER value from each well.; values are expressed as mean ± standard deviation; AECs: alveolar epithelial cells, TEER: transepithelial electrical resistance.
(TIF)

S8 Fig Effect of Dex on the expression of OCLN and CLDN18. (A) Relative expression of OCLN and (B) CLDN18 mRNA in AECs after 6 h of Dex stimulation, n = 3 (three independent experiments).
(TIF)

S1 Raw image (PDF)
