# Supplementary figures and images for: Dexamethasone restores TNFα-induced epithelial barrier dysfunction in primary rat alveolar epithelial cells
Source: PLoS One. 2023 Dec 27;18(12):e0295684. doi: 10.1371/journal.pone.0295684 (PMC10752552; doi:10.1371/journal.pone.0295684)

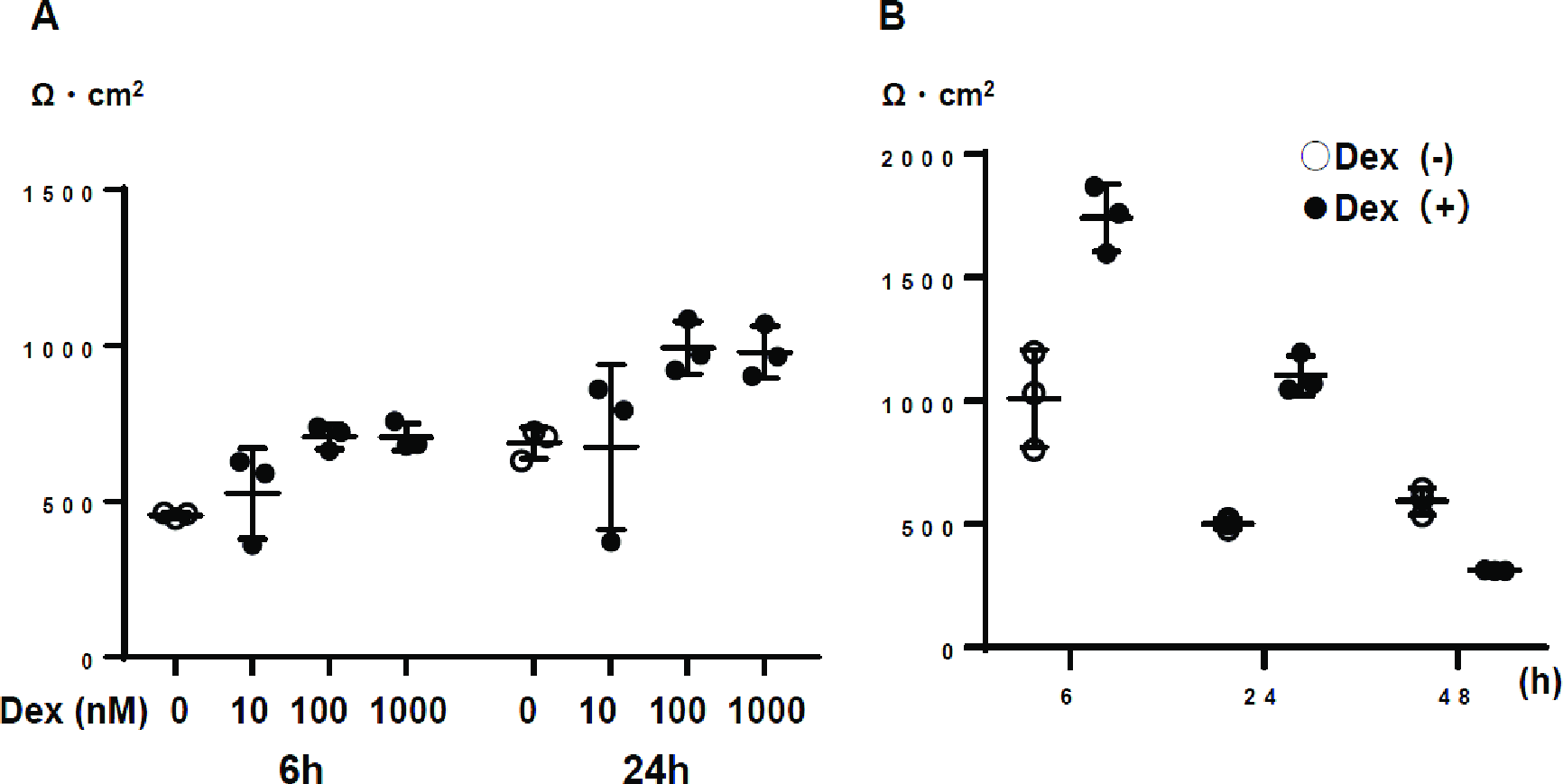

Supplement: S1 Fig — (A) TEER in AECs stimulated using different doses of Dex at 6 and 24 h; (B) Time course of TEER in AECs stimulated using 1,000 nM Dex; n = 1, triplicate wells/experiment, Each dot means TEER value from each well.; values are expressed as mean ± standard deviation; AECs: alveolar epithelial cells, TEER: transepithelial electrical resistance, Dex: dexamethasone. (TIF) [file pone.0295684.s001.tif]

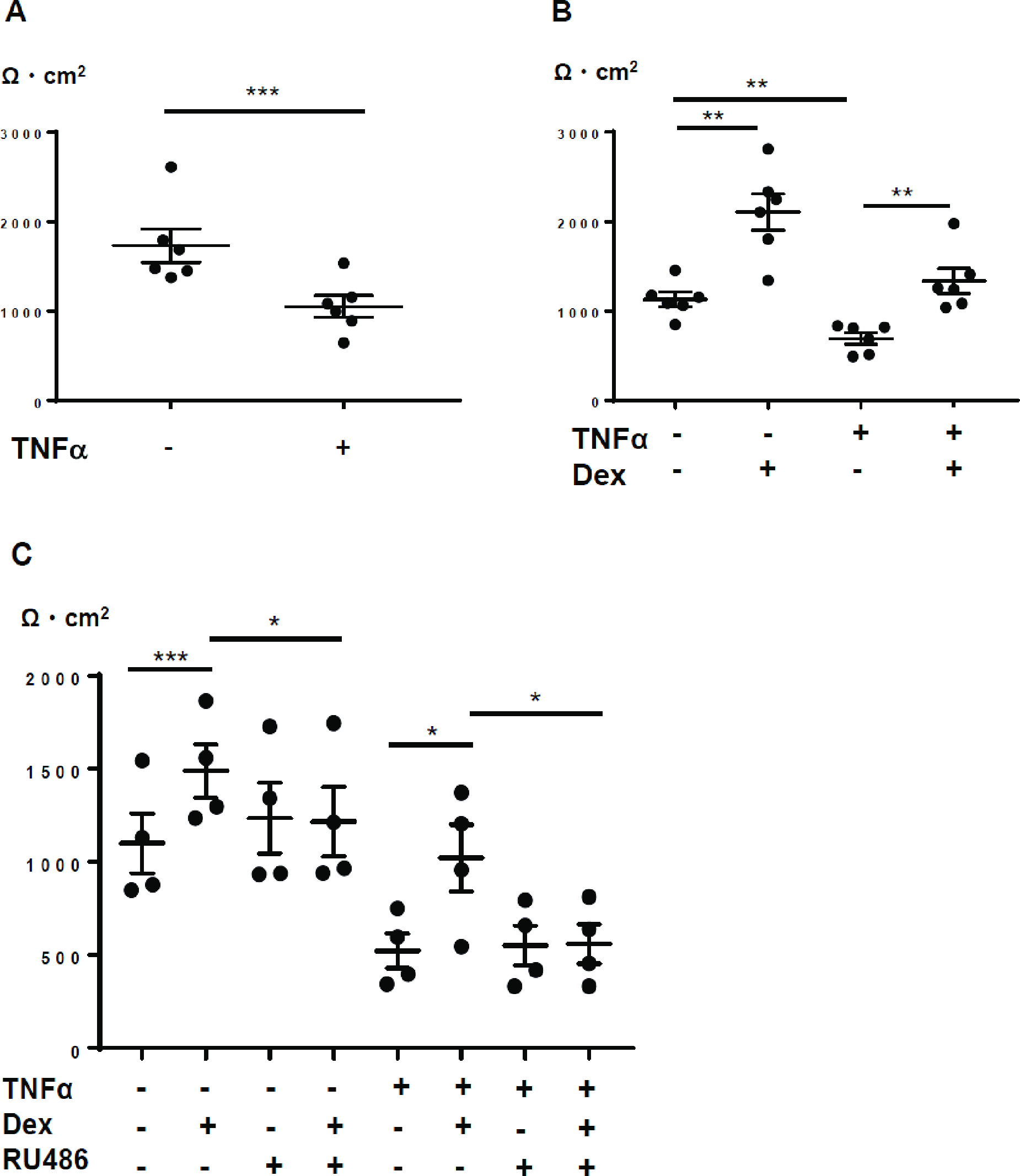

Supplement: S2 Fig — (A) TEER in AECs stimulated with ± 10 ng/mL recombinant rat TNFα for 24 h, n = 6 (six independent experiments). (B) TEER in AECs stimulated with ± 10 ng/mL recombinant rat TNFα for 48 h and ± 1,000 nM Dex for 24 h, n = 6 (six independent experiments). (C) TEER in AECs under treatment of ± 10 ng/mL recombinant rat TNFα for 48h, ± 1000 nM Dex and ± 10 μM RU486 for 24 h, n = 4 (four independent experiments); * p<0.05, ** p<0.01, *** p<0.001; TEER: transepithelial electrical resistance, TNFα: tumor necrosis factor-alpha, Dex: dexamethasone. (TIF) [file pone.0295684.s002.tif]

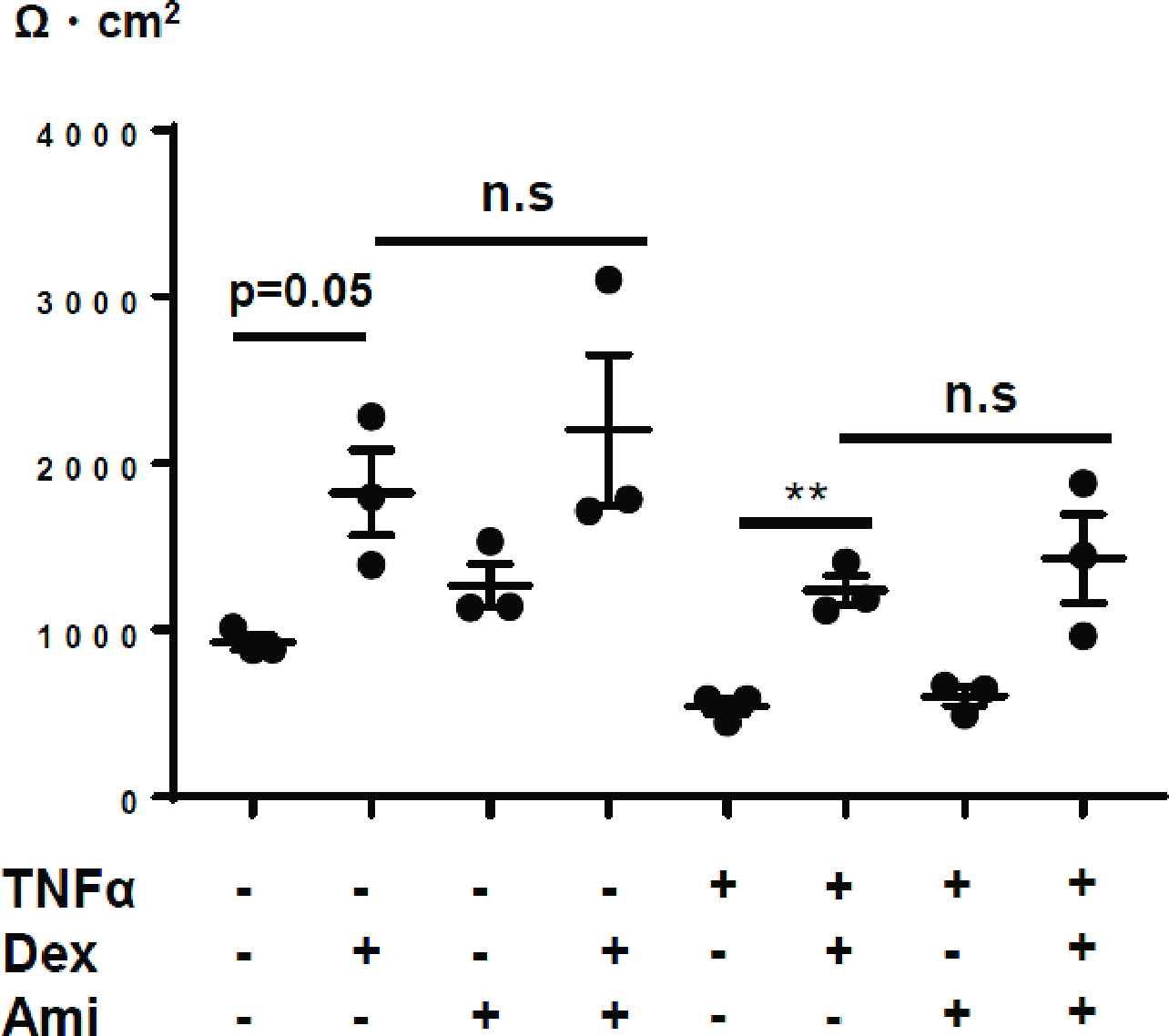

Supplement: S3 Fig — TEER in AECs stimulated using ± 10 ng/mL recombinant rat TNFα ± 1,000 nM Dex ± 10 ng/mL Amiloride after 24 h of Dex exposure, n = 3 (three independent experiments, triplicate wells/experiment, Each dot means the average of triplicate wells.); values are expressed as mean ± standard error of the mean; ** p<0.01; TEER: transepithelial electrical resistance, TNFα: tumor necrosis factor-alpha, Dex: dexamethasone, Ami: amiloride, n.s.: not significant. (TIF) [file pone.0295684.s003.tif]

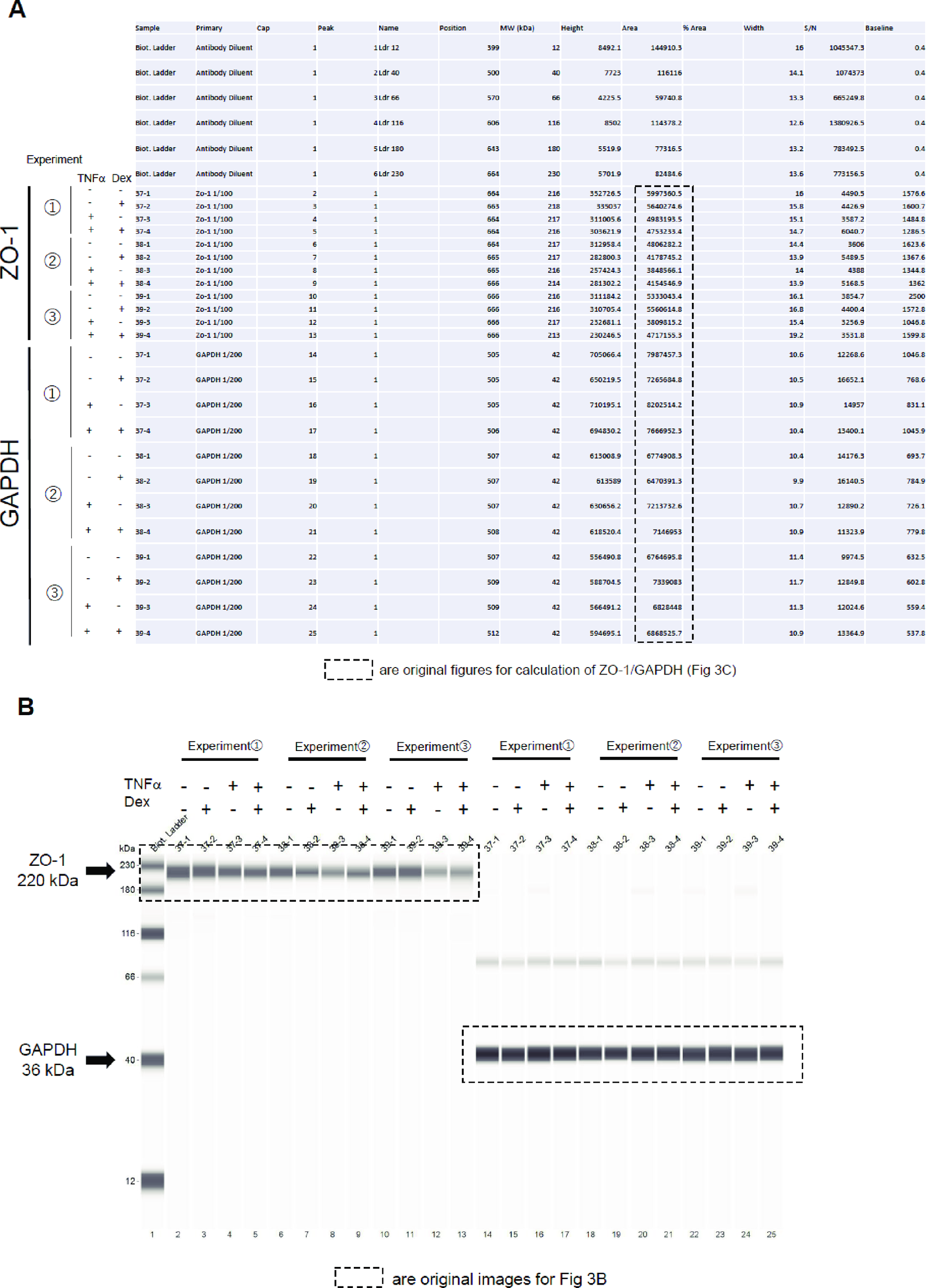

Supplement: S4 Fig — (A) Results visualized as electropherograms representing peak of chemiluminescence intensity and (B) as lane view from signal of chemiluminescence detected in the capillary. (TIF) [file pone.0295684.s004.tif]

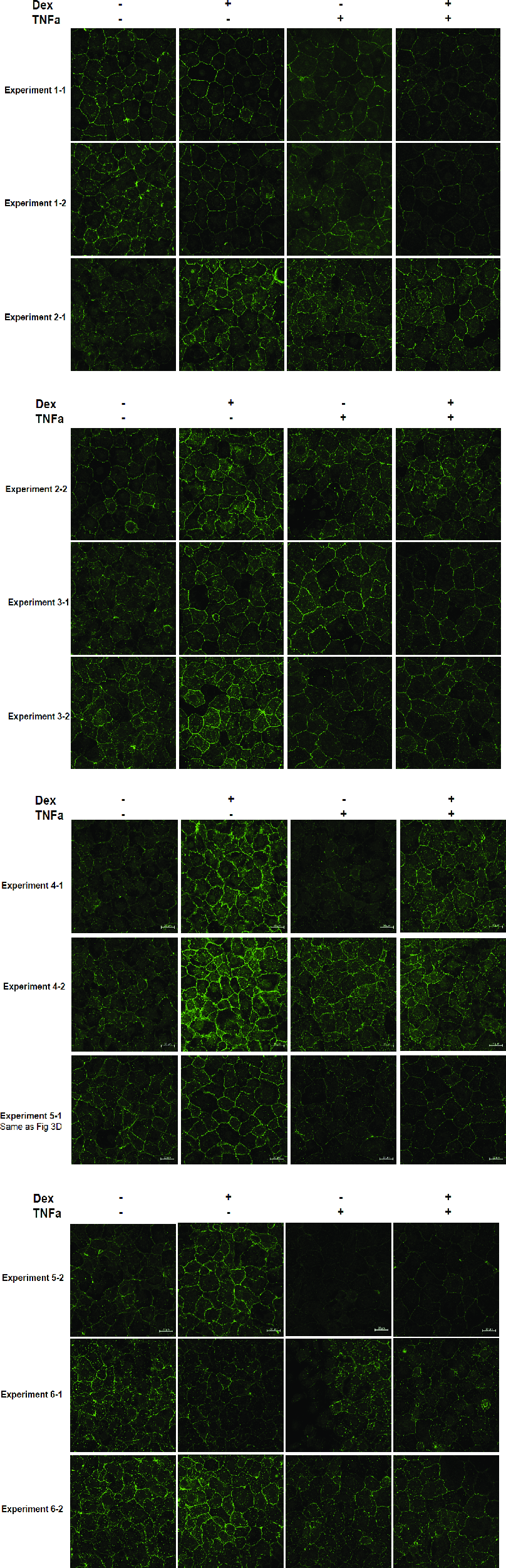

Supplement: S5 Fig — green: ZO-1; magnification: ×400. (TIF) [file pone.0295684.s005.tif]

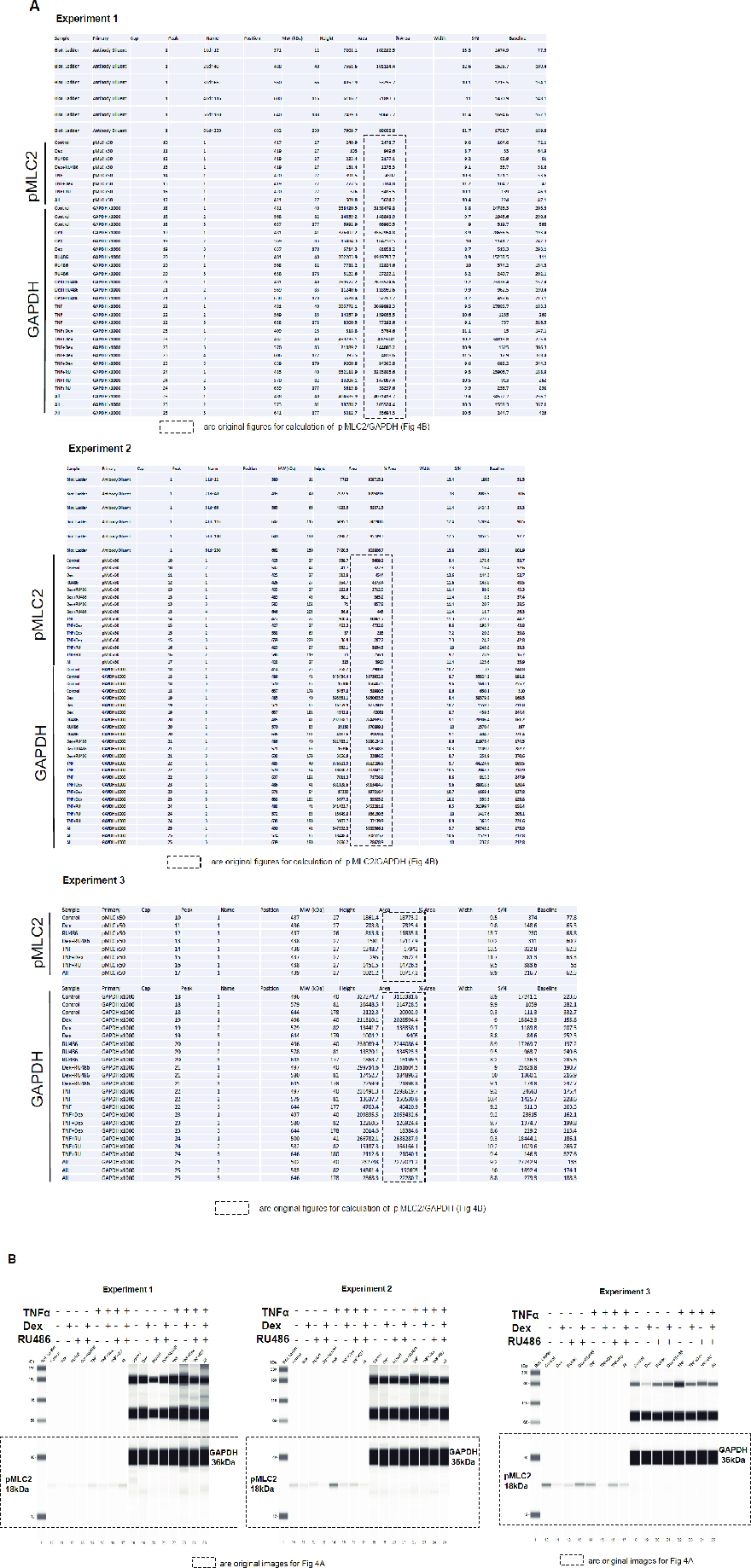

Supplement: S6 Fig — (A) Results visualized as electropherograms representing peak of chemiluminescence intensity and (B) as lane view from signal of chemiluminescence detected in the capillary. (TIF) [file pone.0295684.s006.tif]

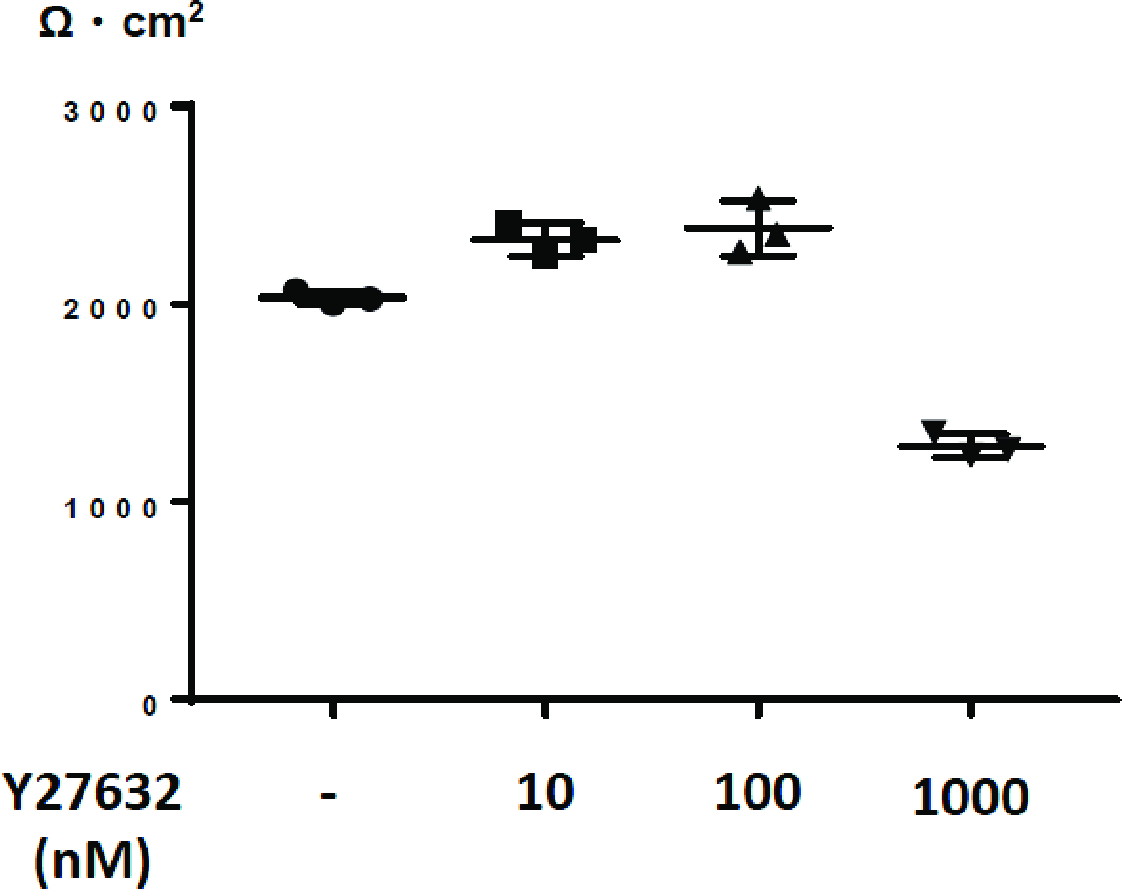

Supplement: S7 Fig — TEER in AECs stimulated using different doses of ROCK inhibitor (Y27632) at 24 h; n = 1, triplicate wells/experiment, Each dot means TEER value from each well.; values are expressed as mean ± standard deviation; AECs: alveolar epithelial cells, TEER: transepithelial electrical resistance. (TIF) [file pone.0295684.s007.tif]

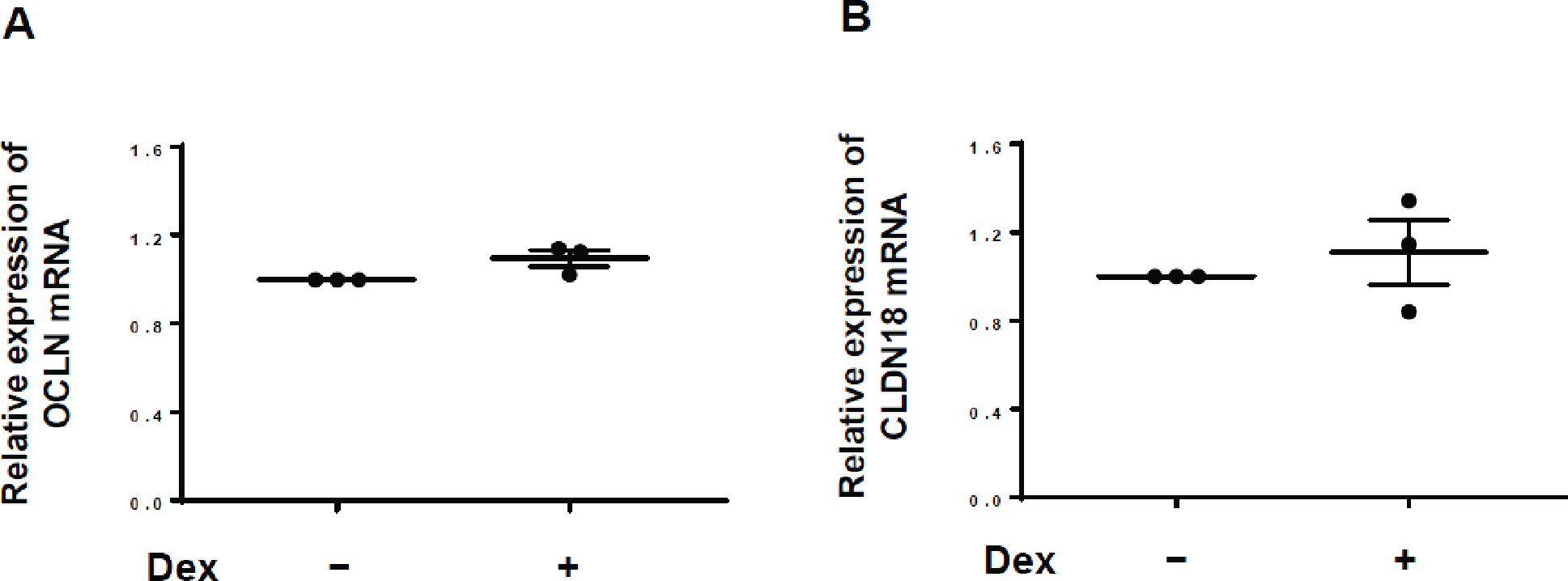

Supplement: S8 Fig — (A) Relative expression of OCLN and (B) CLDN18 mRNA in AECs after 6 h of Dex stimulation, n = 3 (three independent experiments). (TIF) [file pone.0295684.s008.tif]

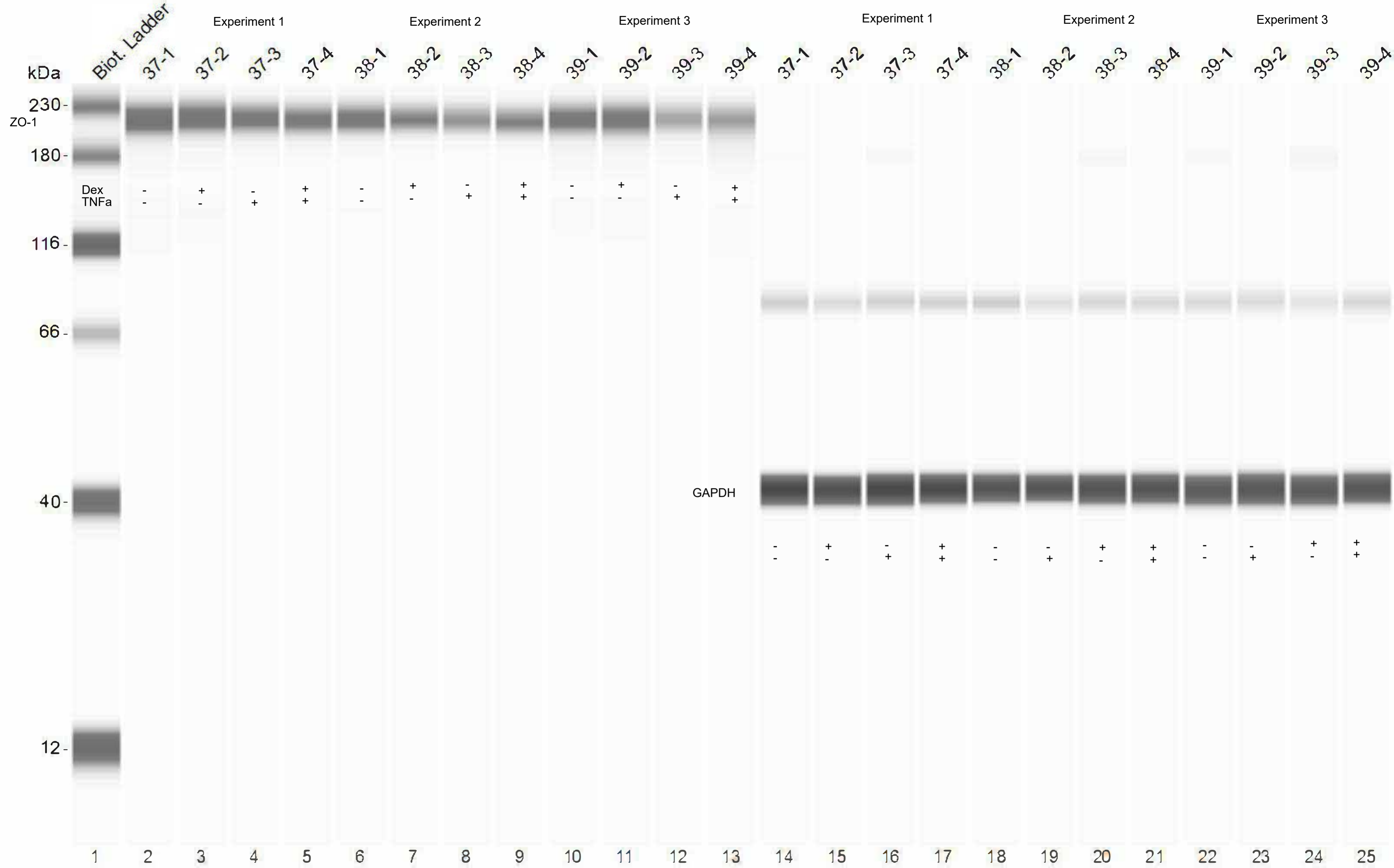

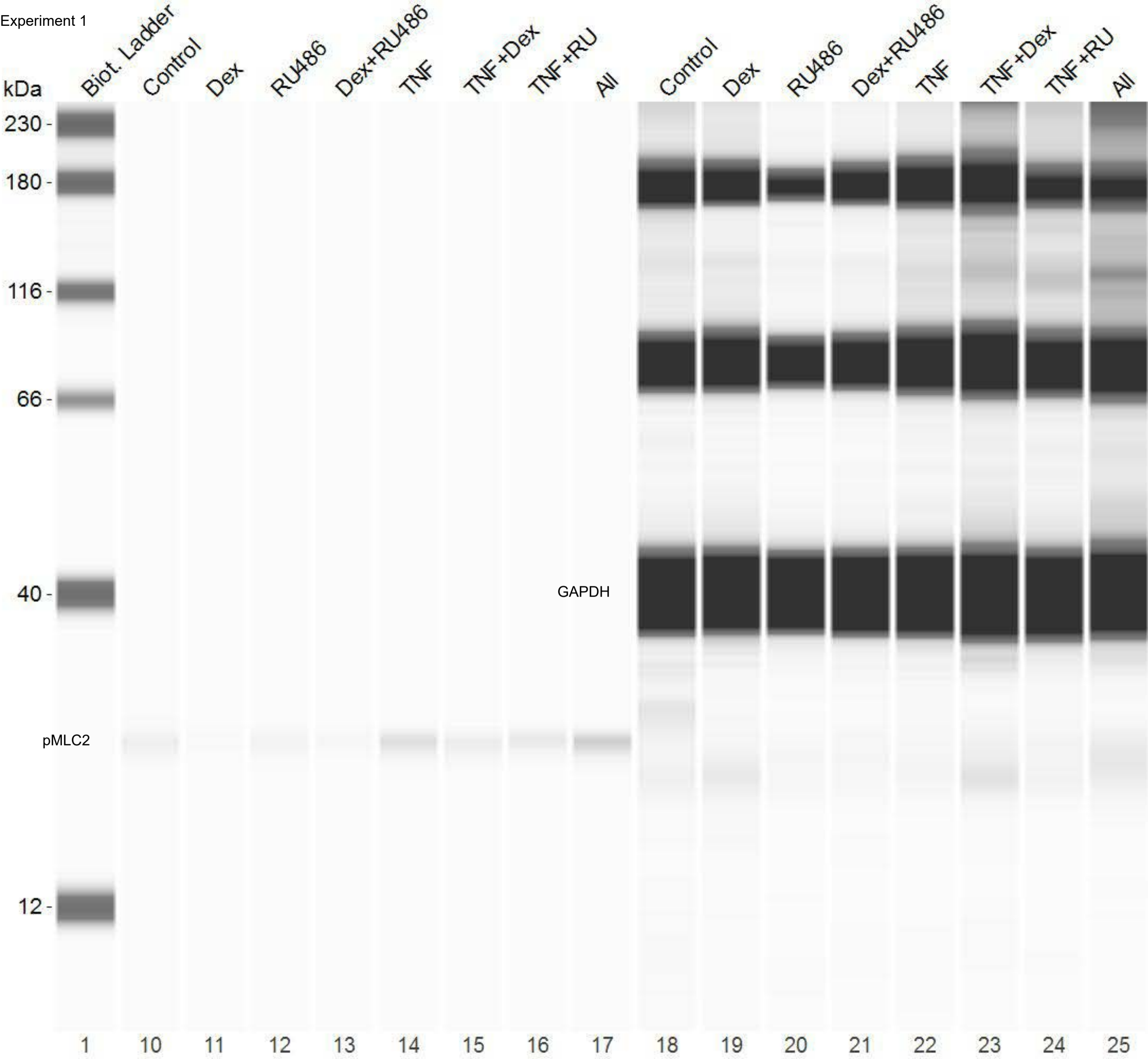

Experiment 2

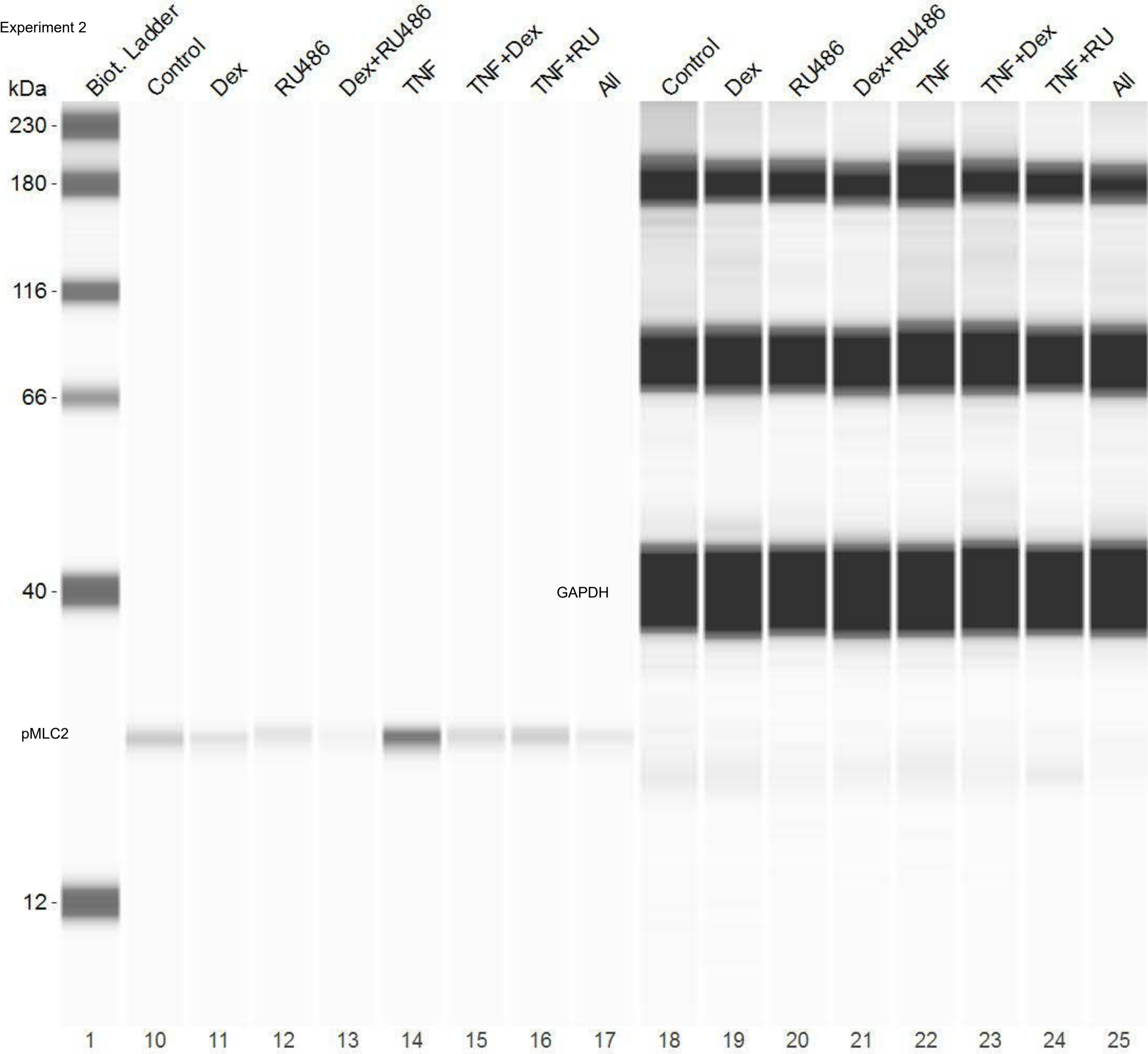

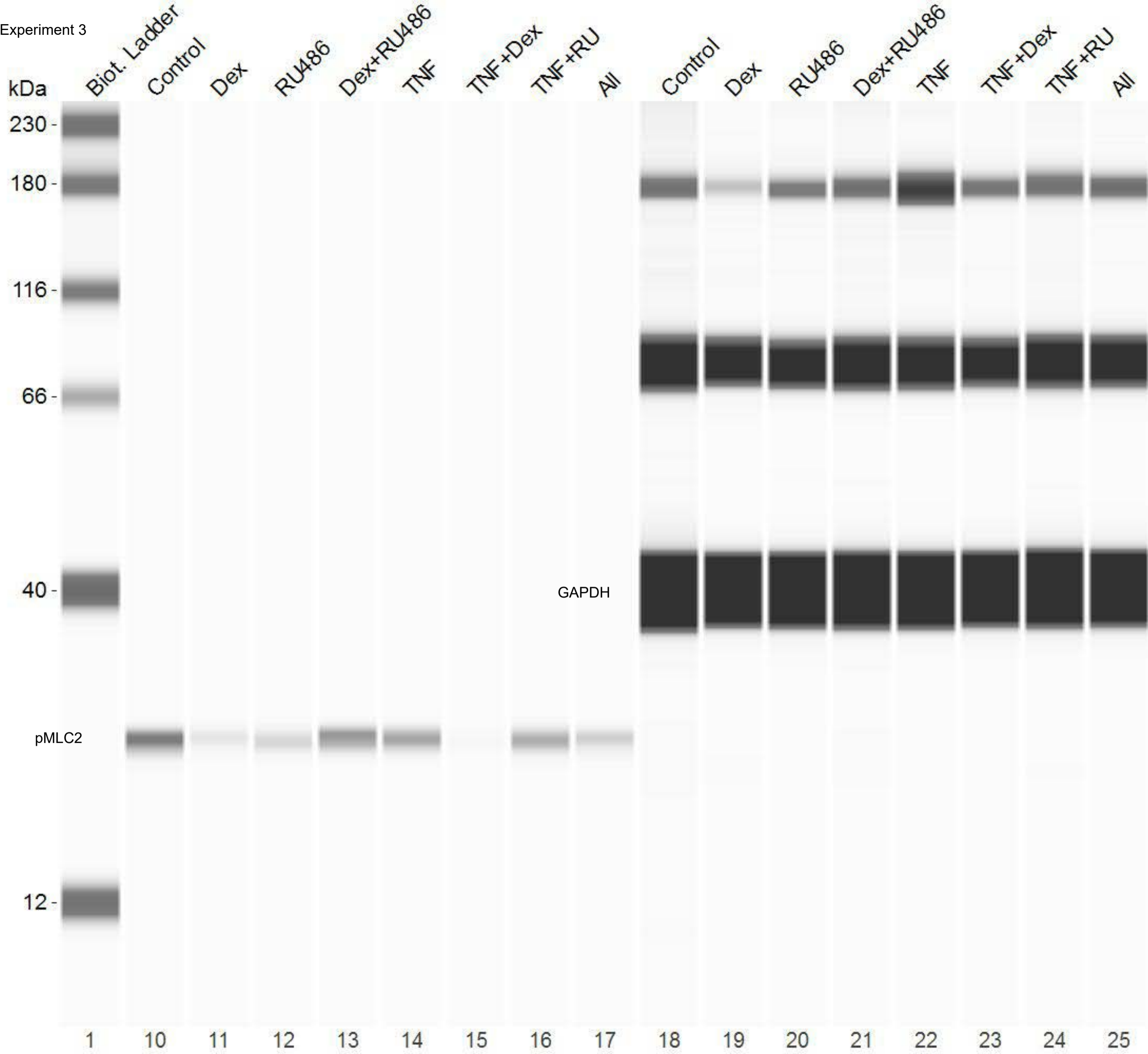

Supplement: S1 Raw image — (PDF) [file pone.0295684.s009.pdf]
